# Special Issue “Emerging AI+X-Based Sensor and Networking Technologies including Selected Papers from ICGHIT 2022–2023”

**DOI:** 10.3390/s24020546

**Published:** 2024-01-15

**Authors:** Byung-Seo Kim, Muhammad Khalil Afzal, Rehmat Ullah

**Affiliations:** 1 Department of Software and Communication Engineering, Hongik University, Sejong 30016, Republic of Korea; 2 Department of Computer Science, Wah Campus, COMSATS University Islamabad, Wah Cantt 47040, Pakistan; khalilafzal@ciitwah.edu.pk; 3 Cardiff School of Technologies, Cardiff Metropolitan University, Llandaff Campus, Western Avenue, Cardiff CF5 2YB, UK; rullah@cardiffmet.ac.uk

This Special Issue is a collection of selected papers from the 10th and 11th International Conferences on Green and Human Information Technology (ICGHITs). The two ICGHITs were held on Juju Island, Korea, on 19–21 January 2022, and in Bangkok, Thailand, on 31 January–2 February 2023, respectively. Reflecting the enormous interest in AI, the conference themes of 10th and 11th ICGHITs were “Emerging Artificial Intelligent (AI)+X technology” and “Hyper Automation + Human AI”, respectively. The ICGHIT is an international conference focusing on green and information technologies focused on humanity. The goal of the ICGHIT is to form an interdisciplinary platform for the advancement of green technology and human-related IT. The main topics of the conference are green information technology (IT), communication and the Internet of Things (IoT), computer and network security, multimedia and signal processing, control and intelligent systems, and green IC system design. Many papers on advanced technology were presented and various products from multiple companies were exhibited. The conference hopes to provide the possibility for technical exchange and advancement, cooperation among the participants, and local development in the country in which the conference is held. The organizing committee members believe that green technology and human IT can work synergistically to enhance human welfare and humanity in the present and future technological society. The philosophy of the conference is the achievement of the goal of IT, that is, to improve human welfare and happiness. Therefore, the ICGHIT emphasizes humanity as well as technology. 

We selected 21 papers from the ICGHITs 2022 and 2023 for this Special Issue and 10 papers have been accepted through a rigorous peer review process. The contributions are listed below.

Reflecting the current emerging technologies for humans, the themes of both of conferences focused on AI technology. It can clearly be observed that AI, including machine learning (ML) and deep neural networks (DNNs), is being quickly and deeply incorporated into our daily lives. Furthermore, emerging AI technologies also greatly impact research activities in many areas. As shown in contributions 2, 3, 4, 7, 8 and 9, this trend is also present in the selected papers in this Special Issue, in which AI-based methods are applied to various research areas including network security, routing protocols, signal detection and clustering mechanisms. 

Even though secure and strong authentication is considered an important function for communications, there are still security issues in authentication. As one authentication method, Personal Identification Numbers (PINs) are well and widely used. Nevertheless, generating and remembering unique and secure passwords is difficult for users. In particular, this issue has become more pronounced as people are increasingly using secure applications like mobile finance applications in their daily life. To solve this issue, Nabeela et al. (contribution 1) proposed a method to generate usable and secure passwords by integrating graphics and arithmetic operations, named GRA-PIN (GRAphical and PIN-based). In the proposed method, users must select four different options to create a password: a selection of two-digit numbers, selection of one secret image, selection of swipe-up/down position for arithmetic operation, and selection of the password position in the final four-digit PIN. The user must also provide a secret answer in case of forgetting their password. Every time the user logs on, a new password will be generated, which makes it secure against shoulder surfing, guessing and camera attacks.

Even though secure routing protocols over ad hoc mobile networks have been studied over recent decades, there are still many issues to be resolved. In the article by Pramitarini et al. (contribution 2), sinkhole attacks in vehicle ad hoc networks (VANETs) are studied and a hybrid-price auction-based secure routing (HPA-SR) protocol is proposed. The protocol utilizes hybrid auctions with a Markov decision process (MDP) to establish a secure route from a source node to a destination node. It has two steps: clustering and routing. In the clustering step, by using the advanced node speed and cosine similarity, all nodes are clustered. In the routing step, each node is used in the MDP to conditionally select which kind of auction method establishes a secure route against the sinkhole attack. Through extensive evaluation works, it was shown that the proposed method outperforms the AODV+ASCS, HPA-SR, and AODV protocols in terms of security and the packet delivery ratio.

A novel method in the article by Rahman et al. (contribution 3) was proposed to enhance the estimation of the distance between RFID readers and tags with an electronic steering parasitic array radiator (ESPAR) antenna. The proposed method uses the beam scanning method (BSM) to fix the exact location of the RFID tags along with its particular direction. The accuracy of the BSM for shorter communication distances (≥25 m) is very high compared with other methods. The beam steering of the ESPAR antenna or an array of ESPAR antennas exhibits a very good performance regarding radiation patterns, gains (8.17 dBi, 11.40 dBi) and reflection coefficients. 

Due to the increased use of Internet of Things devices, home appliances account for a large portion of smart homes’ energy consumption. Energy optimization is a key challenge in smart homes. Therefore, Mehmood et al. (contribution 4) proposed an optimization technique that addresses the trade-off between energy saving and user convenience, considering the use of air pressure, dew point and wind speed. The authors used the gray wolf optimizer and particle swarm optimization algorithms. Long short-term memory recurrent neural networks were designed to predict the appliances’ energy use. The results indicated that the proposed model performs better in terms of root mean square error and optimizes energy consumption.

Blockchain technology is an information security solution that functions on a distributed ledger system. Blockchain technology has significant potential for securing the Internet of Things. However, the integration of the Internet of Things and blockchain technologies leads to a number of issues. One of the most important challenges is the energy consumption of different blockchain algorithms. Internet of Things devices are typically low-powered devices; thus, the energy consumption of any blockchain node must be kept low. Arachchige et al. (contribution 5) analyzed the correlation between the blockchain temperature and energy consumption data based on three blockchain algorithms to compare variations in energy requirements. Triwidyastuti et al. (contribution 6) found that the temperature of Internet of Things devices and their energy consumption are highly correlated, suggesting that there is a need for the development of energy-efficient blockchain algorithms. 

Triwidyastuti et al. studied the performance of multi-hop transmission regarding secrecy for wireless sensor networks under various eavesdropping attacks. To improve the performance, the authors proposed two node-selection schemes in each cluster, namely minimum node selection and optimal node selection, and derived a closed-form expression for the secrecy outage probability under different eavesdropping attacks. The results reveal that an active eavesdropping attack is more destructive compared to a passive attack since an active eavesdropper generates a jamming signal. In addition, in contrast to minimum node selection, the optimal node-selection scheme has a high complexity and a significantly improved performance in terms of secrecy.

In the article by Triwidyastuti et al. (contribution 7), a deep learning algorithm is applied to secure a multicasting routing protocol in flying ad hoc networks with cell-free massive MIMO, referred to as “deep learning-based secure multicast routing (DLSMR)”. DLSMR particularly focuses on wormhole attacks in the multicasting routing protocol. The DLSMR protocol predicts whether the route is secure based on network information such as the node ID, distance, destination sequence, hop count and remaining energy. To improve the node connectivity and manage multicast members, the authors also proposed a top-down particle-swarm-optimization-based clustering (TD-PSO) protocol to maximize the cost function. The node degree, cosine similarity, cosine distance and cluster head energy were considered to guarantee convergence to the global optimum. Through extensive simulation works, the proposed DLSMR+TD-PSO protocol exhibits better scalability and effectively improves the PDRs, routing delays and control overheads compared to other related methods.

Similar to the previously mentioned contribution 7, Amalia et al. (contribution 8) also applied a deep learning algorithm to resolve security issues in routing protocols, but in a different application to vehicle ad hoc networks. The authors propose two protocols: a deep-learning-based secure routing (DLSR) protocol and a deep-learning-based clustering (DLC) protocol. The former is used to set up secure routing by detecting abnormal nodes utilizing deep learning at each node, and the latter is used to enhance the connectivity and reduce the overheads by using a DNN. While the DLSR protocol considers the remaining energy, distance and hop count as parameters, the DLC protocol considers the cosine similarity, the cosine distance and the node’s remaining energy. 

Deep learning was applied to detect low-probability-of-intercept (LPI) radar signals. By taking into account the repeated-pulse characteristics of radar signals and utilizing a DL model based on long short-term memory (LSTM), contribution 9 utilized the periodic autocorrelation function (PACF) to provide in-depth information about LPI radar signals. The proposed model exhibits a better detection probability over various channels with different signal-to-noise ratios than convolutional neural network (CNN)-based detection methods.

Park et al. (contribution 10) propose a solution to secure blockchain systems against distributed denial of service (DDoS) attacks. Particularly, they focus on early DDoS attack detection by utilizing polynomial regression with blockchain data. In addition to this method, the authors propose an alternative detection method by employing statistical analysis, specifically using the coefficient of determination, to enhance the accuracy. The first method exhibits a mean square error (MSE) of 0.0215 and the alternative method exhibits an accuracy of 0.8667 in detecting DDoS attacks.

